# Impact of a multidisciplinary team for the management of thrombotic microangiopathy

**DOI:** 10.1371/journal.pone.0206558

**Published:** 2018-11-02

**Authors:** Miguel G. Uriol Rivera, Sheila Cabello Pelegrin, Carmen Ballester Ruiz, Bernardo López Andrade, Javier Lumbreras, Aina Obrador Mulet, Albert Perez Montaña, Mireia Ferreruela Serlavos, José Ignacio Ayestarán Rota, Joana Ferrer Balaguer, Olga Delgado Sanchez, Lucio Pallares Ferreres, Antonio Mas Bonet, María Jose Picado Valles, Rosa María Ruíz de Gopegui Valero

**Affiliations:** 1 Nephrology Department, Son Espases University Hospital, Palma de Mallorca, Spain; 2 Hematolgy Department, Son Espases University Hospital, Palma de Mallorca, Spain; 3 Pediatric Nephrology Unit, Son Espases University Hospital, Palma de Mallorca, Spain; 4 Intensive Care Unit, Son Espases University Hospital, Palma de Mallorca, Spain; 5 Inmmunology Department, Son Espases University Hospital, Palma de Mallorca, Spain; 6 Pharmacy Department, Son Espases University Hospital, Palma de Mallorca, Spain; 7 Autoinmmune Diseases Unit, Son Espases University Hospital, Palma de Mallorca, Spain; 8 Neuroradiology Unit, Son Espases University Hospital, Palma de Mallorca, Spain; 9 Department of Gynecology and Obstetrics. Son Espases University Hospital, Palma de Mallorca, Spain; Azienda Ospedaliero Universitaria Careggi, ITALY

## Abstract

**Background:**

Thrombotic microangiopathy (TMA) is an important complication associated with several diseases that are rare and life-threatening. TMA is common to thrombotic thrombocytopenic purpura (TTP) and hemolytic uremic syndrome (HUS). TTP is defined by a severe deficiency of ADAMTS13, and early treatment is associated with good prognosis. The diagnosis of HUS can be difficult due to the potential multiple etiologies, and the best treatment option in most cases is not well-established yet. The implementation of a multidisciplinary team (MDT) could decrease the time to diagnosis and treatment for HUS and may improve the outcomes of these patients.

**Objective:**

To determine the impact of MDT on morbidity and mortality [death or chronic renal replacement therapy (CRRT) requirements], incidence and response time [(RT) defined as the period between hospital admission and the first day of direct therapy administration], length of stay at an intensive care unit (ICU-LOS) and total hospitalization (T-LOS) were also assessed.

**Methods:**

We compared a pre-MDT implementation period (from January/2008 to May/2016) versus post-MDT period (from May/2016 to December/2016). The screening TMA diagnosis was made according the following criteria: hemolytic anemia, thrombocytopenia and acute renal damage and without ADAMTS13 deficiency. An online chat was implemented to provide instant medical information.

**Results:**

Twenty-eight patients were included. The incidence changed from 2.3 cases/pre-MDT: (all cases: n = 18) to 10 cases/year post-MDT (all cases: n = 10). Two patients died in pre-MDT and post- MDT (11% versus 20%, *P* = 0.60). From pre-MDT, the number of patients who required CRRT by post-MDT decreased from 7 (39%) to 0, *P* = 0.03. Similarly, RT, ICU-LOS and T-LOS [median(p25-p75)] decreased from 10 (2–12) days to 0.5 (0–1.5) days, *P* = 0.04, from 16 (9–30) days to 10 (4–13) days, *P* = 0.01 and from 33 (22–53) days to 16 (12–32) days, *P* < 0.01, respectively.

**Conclusion:**

MDT implementation was associated with a greater number of patients who meet TMA criteria. A decrease in the RT and T-LOS periods were observed and associated with better outcomes in these patients.

## Introduction

Thrombotic microangiopathy (TMA) is a syndrome characterized by hemolytic anemia, thrombocytopenia and acute multiple organ failure, especially renal and/or neurologic organs, with fatal consequences if the diagnosis and treatment are not performed early. The main differential diagnostic possibilities include thrombotic thrombocytopenic purpura (TTP), which is defined by ADAMTS13 deficiency [1–3] [(*a disintegrin and metalloprotease with thrombospondin type 1 domains*, *member 13 of the family)* and hemolytic uremic syndrome (HUS) with multiple potential etiologies [4].

Several pathological conditions, drugs and infections may facilitate the development of TMA as a complication, a circumstance that worsens the prognosis of the underlying disease. Some conditions may also unmask congenital alterations of the complement system [5–7]. Moreover, it often overlaps with signs and symptoms of other diseases, making diagnosis of TMA difficult, which could delay treatment administration.

Therapeutic plasma exchange (TPE) and steroid administration have dramatically changed the otherwise grim prognosis of TTP [8], while the utilization of eculizumab (a complement inhibitor) is the first line treatment for complement disorders-HUS (CD-HUS) [9, 10]. However, most patients are affected by secondary TMA, and in those cases, the optimal treatment is not well established [11].

TMA requires urgent identification and treatment because of the high mortality rate, and irreversible end-organ damage in untreated patients has been observed. Complex care is needed for the majority of patients. In many cases, this care includes TPE, renal replacement therapy, immunosuppressive drugs and immunological and laboratory tests.

The implementation of a multidisciplinary team (MDT) for the management of other conditions including cancer, diabetes and other diseases has shown benefits. The rationale for introducing multidisciplinary teams for TMA management is that as the management of disease becomes more complex, it is important to involve all key professional groups in making urgent clinical decisions for individual patients and the need to improve the prognosis and understanding of this serious disease. In this study, we evaluated the effect of an MDT implemented for the diagnosis and treatment of TMA on clinical outcomes and average hospitalization length.

## Materials and methods

To address this research goal, the investigators designed and implemented a retrospective study to evaluate the potential differences between pre-MDT and post-MDT implementation at a tertiary referral center in Palma de Mallorca between January 2008 and December 2016. The etiology was predominantly TMA infection-related; other details are shown in Table 1.

**Table 1:** Thrombotic microangiopathy etiologies.

N = 28	
***Etiology*:**	
**Malignancies, n (%)**	**3 (10.7)**
**Drug-associated, n (%)**	**3 (10.7)**
**Autoimmune disease, n (%)**	**2 (7.2)**
**Infectious**	
• ***Pneumococci*, *n (%)***	**2 (7.2%)**
• ***Others*, *n (%)***	**4 (14.2%)**
• ***STEC-HUS*, *n (%)***	**2 (7.2)**
**Glomerulonephritis, n (%)**	**2 (7.2)**
**Malignant hypertension, n (%)**	**2 (7.2)**
**Kidney transplant rejection, n (%)**	**1 (3.6)**
**Complement disorders, n (%)**	**5 (17.8)**
**Pregnancy, n (%)**	**2 (7.2)**

**HUS,** hemolytic uremic syndrome; **STEC,** Shiga toxin-producing Escherichia coli.

**Fig 1:**
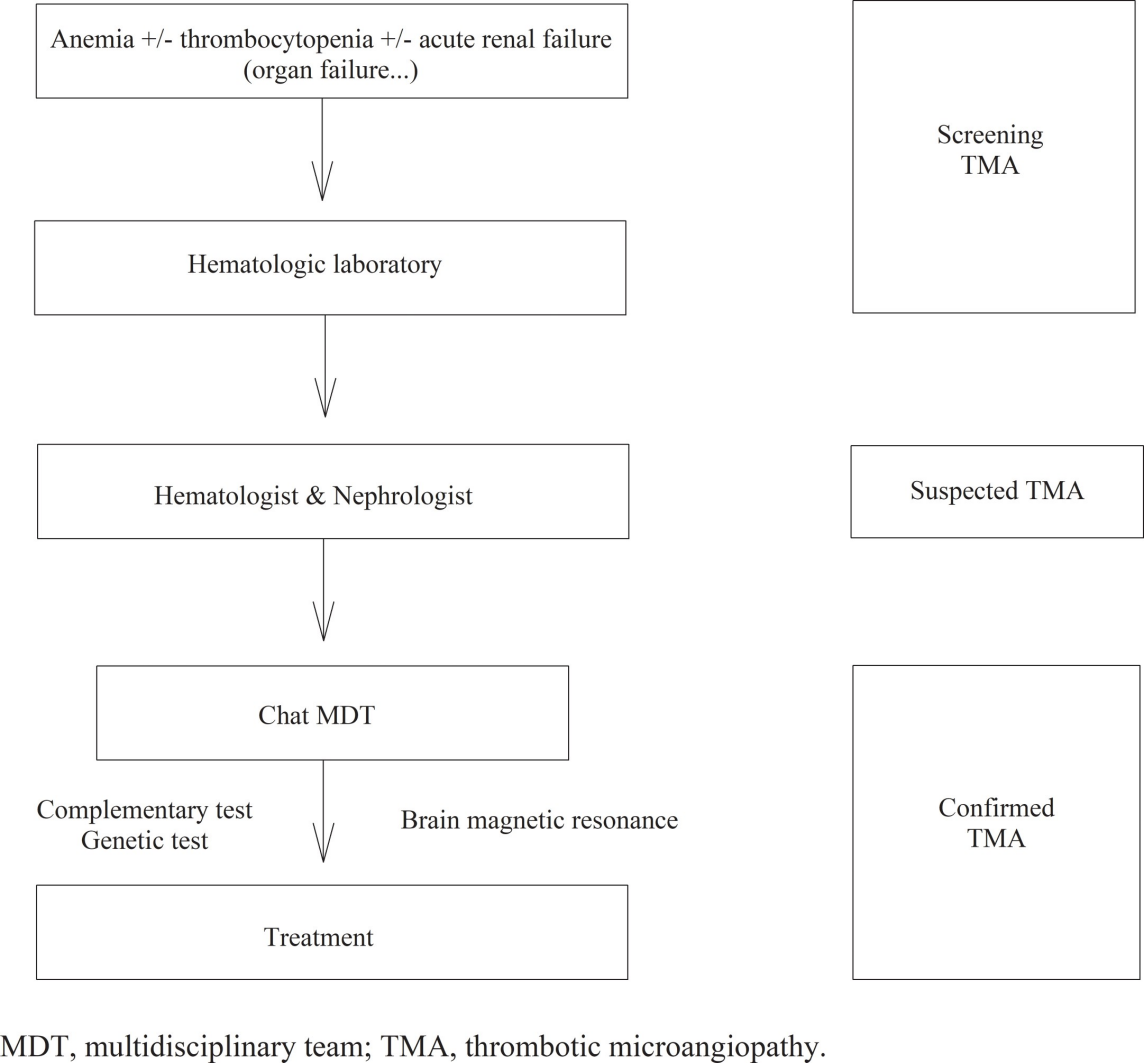
Multidisciplinary team workflow.

**Fig 2:**
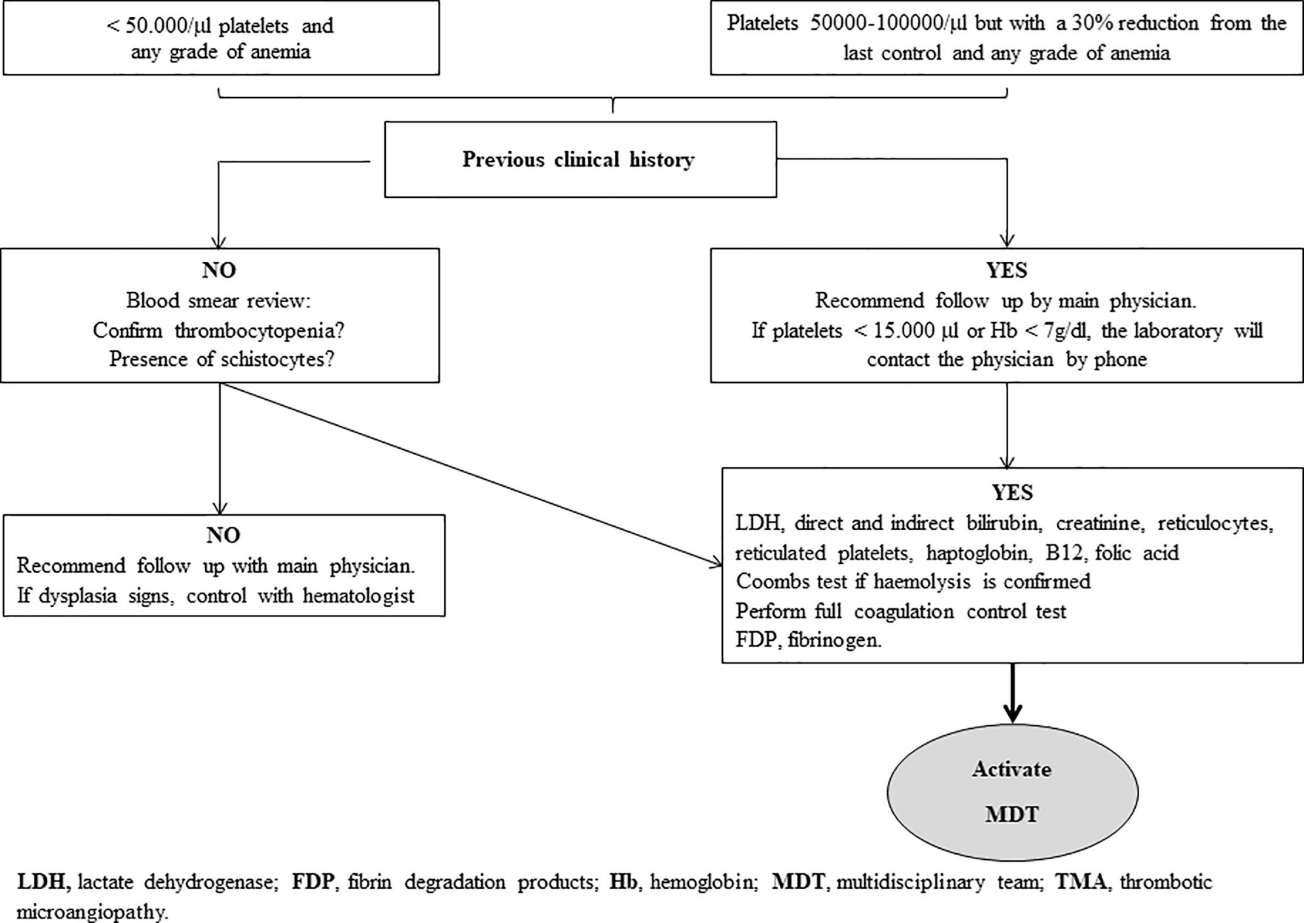
Algorithm for laboratory diagnosis of TMA.

The MDT was created in May 2016 as a working group of physicians specializing in TMA, and they recommended the following criteria for the diagnosis of TMA that are consistent with the published guidelines: i) hemolytic anemia, ii) thrombocytopenia and iii) acute organ failure [1, 12–15], iv) patients without ADMTS13 deficiency. The designed workflow is shown in Fig 1. The screening evaluation at the hematologic laboratory is shown at Fig 2. The protocol treatment included 125 mg /day intravenous steroids (3 consecutive days) and diary TPE (the average plasma exchange per patient was 1.5 plasma volume replacement units). For the following conditions at the time of presentation, anuria, hemodialysis needs, or multiorgan damage, a c5-inhibitor treatment with eculizumab was recommended as a first choice of treatment. Eculizumab was administered as rescue treatment in those patients without clinical or analytical response after three days on TPE.

The multidisciplinary team was formed by hematologists, autoimmunologist, radiologists, obstetricians, clinical pharmacists, immunologists, intensivists, pediatric nephrologists and nephrologists.

A smartphone *chat app* was implemented with the objective of instantly sharing information about the TMA-affected patients, of elaborating the differential diagnoses and of recommending the best treatment option for every case.

### Objectives

Primary Objective: To evaluate the number of patients per year diagnosed with TMA at pre-MDT and post-MDT implementation.

Secondary Objective: To compare the differences between pre-MDT and post-MDT implementation regarding the following outcomes:

- The percentage of patients who died.
- The percentage of patients with CRRT needs.
- Response time (RT): period (days) between hospital admission and the first day of direct therapy administration.
- The length of stay (LOS) in the intensive care unit (ICU) and total LOS (T-LOS).

### Statistical analysis

Data are presented as the mean ± standard deviation (SD) or confidence interval (95%CI), median (P25-P75), or raw numbers (percentage) as needed. The baseline characteristics of both treatment groups were compared using *t-*tests or Mann-Whitney *U*-tests for continuous variables. Normality was assessed using the Kolmogorov–Smirnov test. The differences between both treatment arms in categorical variables were analyzed using the χ^2^ or Fisher exact tests, as appropriate. A Mann-Whitney *U*-test was run to determine if there were differences in LOS between pre-MDT and post-MDT implementation. The data were obtained from the patients’ electronic medical records. Statistical analysis was performed using Statistical Package for the Social Sciences software version 21.0 for Windows. *P* < 0.05 was considered statistically significant.

### Compliance with ethical standards and conflict of interest

The authors declare that they have no conflicts of interest. All procedures performed in studies involving human participants were conducted in accordance with the ethical standards of the institutional and/or national research committee and with the 1964 Declaration of Helsinki and its later amendments or comparable ethical standards. Patients’ records were deidentified and analyzed anonymously.

## Results

The two periods of time evaluated were from January/2008 to April/2016 (pre-MDT) and from May/2016 to December/2016 (post-MDT). Twenty-eight patients met the TMA diagnostic criteria. The mean age was 36 ± 20 (95%CI: 28–44) years. Four (14%) patients were pediatric patients. Eighteen patients (64%) were females. The most common cause of TMA was infection. The basal characteristics are shown in Table 2. The proportion of patients admitted at the ICU was higher at the post-MDT compared to pre-MDT implementation period, but no other basal differences between the two periods evaluated were observed (Table 2).

**Table 2:** Comparison of baseline characteristics between the two-study groups.

Baseline	Pre-MDT (N = 18)	Post-MDT (N = 10)	*P*
**Age (years)**	**36 ± 11**	**34 ± 28**	**0.88**
**Sex, M/F, n (%)**	**6(33) / 12(67)**	**4(40) / 6(60)**	**0.72**
**Hb (g/dL)**	**9.0 ± 2.1**	**10.8 ± 2.3**	**0.06**
**Platelets (1000/μL)**	**44600(23000–84000)**	**39900(19600–84000)**	**0.89**
**LDH (U/L)**	**1994 ± 1425**	**2170 ± 1495**	**0.77**
**Schistocytes (Yes/No), n (%)**	**12(67) / 6(33)**	**9(90) / 1(10)**	**0.36**
**Schistocytes per field**	**3(2–8)**	**4(2–5)**	**0.60**
**HD-ad (yes/no), n (%)**	**10(56) / 8(44)**	**8(80) / 2(20)**	**0.19**
**ICU-ad, (yes/no), n (%)**	**9(50) / 9(50)**	**9(90) / 1(10)**	**0.03**

**Hb,** hemoglobin; **HD-ad,** hemodialysis at admission time; **ICU-ad**: admission at intensive care unit, **LDH:** lactate dehydrogenase; **MDT**: multidisciplinary team

### Incidence

During the pre-MDT period (8 years), 18 patients were diagnosed (2.3 patients/year), while during the post-MDT period (≈ 8 months), 10 patients were diagnosed, which represents a 4x increase over the previous period evaluated.

### Risk of death or chronic renal replacement requirements

Two patients (11%) died in the pre-MDT period (patients died at days 1 and 15 after hospital admission) while another two (20%) died in the post-MDT period (*P* = 0.60).

The diagnostic of TMA in the patients who died at the pre-MDT period was obtained on post-mortem examination. One patient did not have a previous pathologic history, and the other patient had MELD-10 hepatic cirrhosis (model for end-stage liver disease) and early hepatocellular carcinoma.

In the post-MDT period, one patient with basal urologic disease-disseminated malignancy died on the 3^rd^ day after diagnosis, and the other patient had a hematologic malignancy and died a few hours after schistocytes were detected. No treatment for the TMA control in those patients was administered, because futility reasons were considered.

The percentage of patients who required CRRT during the pre-MDT decreased from 7(39%) to 0 (*P* = 0.03) in the post-MDT period.

### Response time

**Fig 3:**
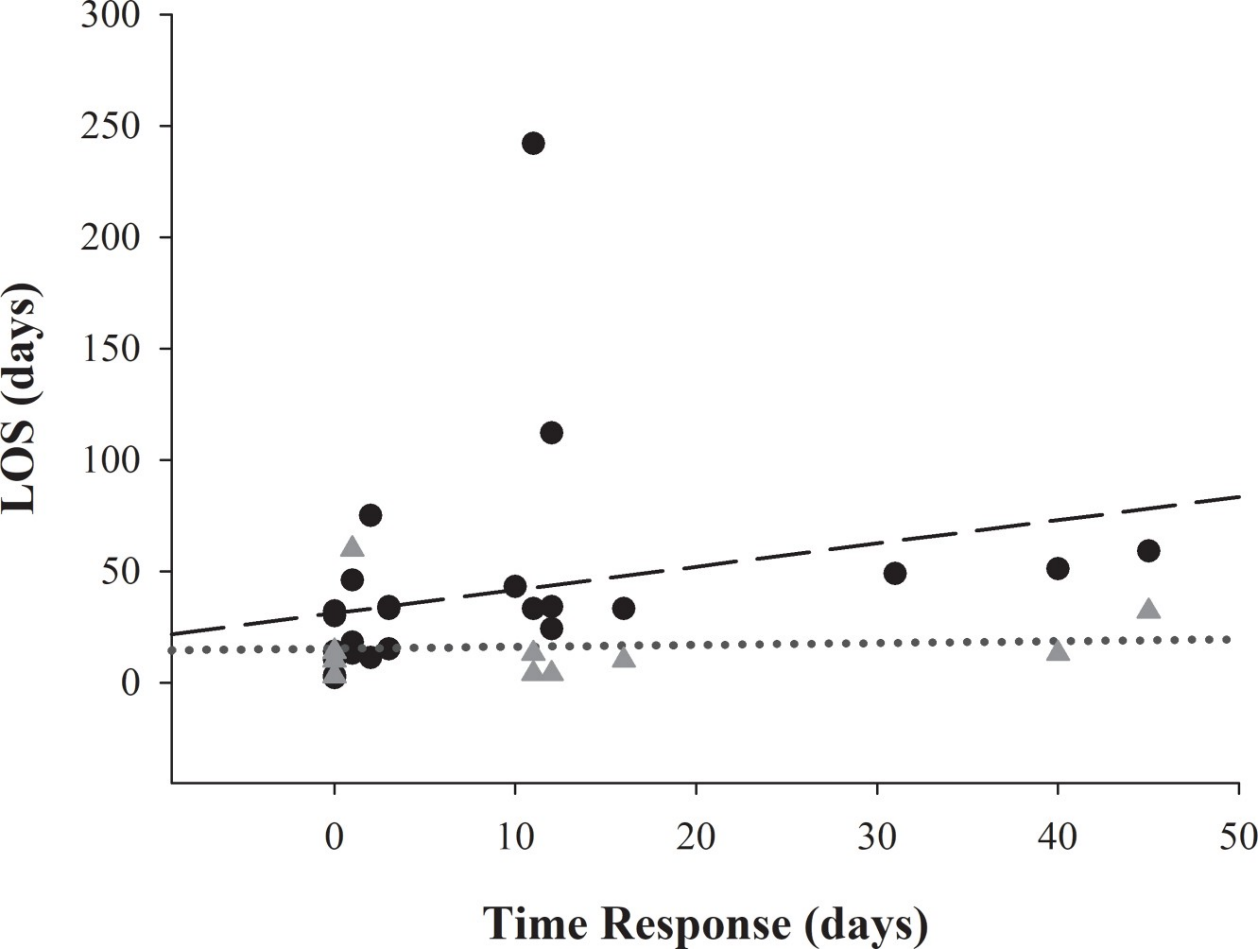
Correlation between time to response (TR) and length of stay (LOS). **Triangles** represent TR and ICU-LOS pairs, and dotted line shows regression line for TR and ICU-LOS (ρ = 0.44; P = 0.06). Circles represent TR and Total-LOS pairs, and long dash shows regression line for TR and Total-LOS (ρ = 0.70; P < 0.01).

The median of RT decreased from 11 days (1.5–12) to 0 days (0–2) (*P* = 0.03) pre-MDT in comparison with the post-MDT period. RT correlates positively with a trend that approached significance with ICU and significantly with T- LOS (Fig 3).

### Length of stay

The median ICU-LOS at pre-MDT and post-MDT decreased from 16 days (7–33) to 11 days (4–18); *P* = 0.05. Similarly, the T-LOS decreased from the pre-MDT [33 days (21–49)] to the post-MDT period [from 33 days (21–49) to 16 days (13–32), *P* = 0.01]. There was a strong positive correlation between TR and T-LOS (ρ = 0.75, *P* < 0.01).

### Treatment

Fourteen (50%) patients received TPE, 9 (50%) and 5 (50%) during the pre-MDT and post-MDT implementation periods, respectively (*P* = 1). Twelve patients received Eculizumab, 5 (28%) pre-MDT and 7 (70%) post-MDT (*P* = 0.03). Eculizumab was indicated as a rescue treatment in 8 patients (3 patients in the post-MDT period). The indications were neurological deterioration plus renal failure (n = 3), neurological deterioration and persistent hemolysis and thrombocytopenia (n = 1), and persistent hemolytic anemia and renal failure (n = 4). Eculizumab was started 13 (7–23) days after the onset of TMA at the pre-MDT and decreased at the post-MDT to 1(0–10) days. TPE was started 3 (2–14) days after the onset of TMA at the pre-MDT and decreased to 0 (0–1) days at the post-MDT.

As a whole, 6 (21%) patients received supported treatment only. Four (%) patients correspond to the pre-MDT period and the other 2 (%) to the post-MDT (χ2: 0.01, *P* = 1.00). The reasons why no direct treatment against TMA and outcomes are explained in Table 3.

**Table 3:** Patients without direct treatment for TMA, features and outcomes.

Patient #	MDT period	Age (years)	Sex	Etiology	Reason for no treatment	Recommended treatment	Outcome
1	Pre	10	M	aHUS, genetic variants of C3 and CFH.	Diarrhea, mild renal dysfunction and rapid renal restauration.	Intravenous fluids.	Normal renal function.
2	Pre	40	F	Pregnancy-HUS.	Mild renal disfunction, low platelets.	Immediate delivery.	Normal renal function, stroke sequelae. Left leg paresis, convulsions.
3	Pre	18	M	Drug related HUS. CNI-BMT.	Mild renal dysfunction, anemia and thrombocytopenia.	Stop CNI.	Normal renal function.
4	Pre	27	M	Urinary infection related-HUS.	Unsuspected TMA. Sepsis suspicion.	Intravenous fluids, antibiotics.	Dead in lower than 20 hours.
5	Post	57	M	Urologic disseminate malignancy.	Futile treatment.	Comfort.	Dead.
6	Post	59	M	Refractory hematologic malignancy.	Futile treatment.	Comfort.	Dead

aHUS: atypical hemolytic uremic syndrome, BMT: bone marrow transplant, CFH: complement factor H, CNI: calcineurin inhibitor, MDT: multidisciplinary team.

### Brain magnetic resonance

The brain magnetic resonance (BMR) was performed by protocol in 7 patients, most within 48 hours of TMA diagnostic. The BMR showed lesions in four patients: hemorrhage (n = 2), micro-bleedings and subdural hematoma (n = 1) and cerebral pseudoatrophy (n = 1). In the other three patients, no alterations in the BMR were observed. The BMR was not performed in three patients, two of them because no active treatment was considered, and they died (already commented previously), and one because made a fast recovery.

### Clinical pharmacy

Our MDT includes a clinical pharmacist who contributes to the team by i) providing medication, ii) discussing general medicine issues, iii) performing literature searches and iv) making a positive contribution to the quality assurance of drug treatment.

### Clinical immunologist

In those patients without a well-identified trigger for TMA, or in those with a previous history of TMA, a diagnosis of complement alternative pathway disorders was evaluated. The immunologist on our team was in charge of managing the samples and facilitated serum values of total hemolytic complement (CH_50_) and complement components C3, and C4. Moreover, autoantibodies associated with a systemic autoimmune disease were rapidly performed.

### Hematology laboratory

The initial screening test is made by hematology laboratory technicians who specialize in the testing and analysis of blood samples. In case of anemia and thrombocytopenia, they report those findings to the hematologist for evaluating the potential TMA affected patient (Fig 2).

## Discussion

We report in this study the beneficial effect of MDT implementation in our hospital, increasing the capacity for TMA detection and decreasing the time to response, LOS and the number of patients with CRRT requirements.

The incidence observed after MDT implementation increased more than 4 times compared to the previous period. This change may be explained by i) the new criteria used as a screening test, which has been shown to be very sensitive and allows the identification of all possible cases; ii) the fact that the most important TMA etiology was infection-related, which is a common condition in a 3^rd^ level hospital; and iii) the hematologic laboratory having a closely integrated working relationship with clinical hematology. We considered that the new diagnostic criteria were able to detect patients affected by TMA who were previously only diagnosed with sepsis. Moreover, this aspect is particularly important since complement disorders have been identified in patients infected by streptococcus pneumonia [5]. That circumstance is especially complicated, not only for the overlapping laboratory test but also for the treatment choice for TMA control during the active infection stage.

Other factors that could be associated with the increased number of patients with TMA are the solid organ and bone marrow transplants. Is essential to describe that TMA associated with hematopoietic stem cell transplant (HSCT) share similar laboratory findings, clinical signs and symptoms (e.g., diarrhoea, skin affection) and the presence of infections due to immunosuppressive therapy, are frequent; so, the diagnosis could be extraordinarily tricky, and treatment delayed whether TMA is not suspected. A recent retrospective cohort study with more than nine hundred HSCT patients evaluated, showed that 13% met TMA-laboratory criteria, and more importantly, the median survival time after the onset of TMA was lower than two months, and only 26% of patients reached on a year of survival [16]. Other potential factors are the use of new potential drugs such as anti-VEGF drugs and anticalcineurin drugs, and patients affected by cancer [17–22], as we found in our study.

Is important to describe that, in our study, the screening performed at the hematologic laboratory detected two cases of TMA that were not prior suspected by their physicians. This condition represents 20% of all cases of TMA detected at the post-MDT. We cannot exclude that due to the overlapping of signs, the presence of TMA could have been undiagnosed, lowering the real incidence of the TMA. Some patients developed cryptic, subclinical TMA or in patient’s cancer-associated TMA, and hematopoietic transplant-associated in whom the laboratory test may help physicians for early TMA identification.

Finally, in the case of suspected TMA-affected patients, the hematologist recognized fine morphological alterations of diagnostic relevance and performed a test extension to confirm microangiopathy hemolytic anemia. This point is crucial in our workflow because it not only accelerates the speed of diagnosis but also facilitates the differential diagnosis.

Of the four patients who died in our study period, in three of them malignancy related TMA was observed. This condition was associated with an aggressive presentation. In the post-MDT period, patients were affected by hematologic and urologic malignancies and received no treatment for TMA due to poor prognosis in the short term. Those patients died at on the 1^st^ and 5^th^ day following TMA diagnosis. All this information suggests that in the patients affected by a malignancy who develop TMA, it could be considered as a lethal complication in a very short period if no direct treatment for TMA is administered [23–25].

Baseline features showed no differences between the two groups, but a higher percentage of patients in the post-MDT period needed dialysis at the time of presentation, and a higher percentage of patients needed admission to the ICU, which indicates the severity of illness in that group. Even with this condition, the percentage of patients who required CRRT decreased from 39% to 0%, which is clinically relevant because the patients were young, and CRRT not only negatively influences survival and quality of life but also increases healthcare costs.

An important decrease in RT in the post-MDT period was observed. The new criteria to facilitate identification of TMA and the screening process at the hematologic laboratory are the main possible reasons for this effect. A direct correlation between RT with ICU-LOS and T-LOS was also observed. Moreover, both ICU and T-LOS decreased in the post-MDT period. We believe that the decrease in RT was not only associated with the reduced hospitalization period, it also positively influenced patient outcomes.

The online chat was a tool used by the MDT that allowed them to i) share medical information at any time; ii) discuss the clinical case; iii) inform team members of the potential needs for ICU admission; iv) request specific diagnostic tests, such as BMR, immunologic test, and ADAMTS-13; and v) recommend personalized treatment for every case. This type of communication not only decreases the time to beginning treatment, but also allows them to indicate what drugs will impact the health system, such as Eculizumab, which ensures a consensus indication with possibly better clinical results.

The utilization of the online chat app is an essential tool to decrease RT. Although we are in an informatized hospital, this type of communication shows clear benefits, providing instant medical information and simplifying diagnostic and treatment recommendations in a real clinical practice.

The MDT is aware of the confidentiality of the medical information shared by this channel. Therefore, sharing data that facilitates identification of the patient being evaluated is avoided as much as possible.

TPE was used in the same percentage of patients over the two periods of time. Nevertheless, a higher percentage of patients were treated with Eculizumab in the post-MDT period. Its early administration was associated with excellent renal prognosis, and no patients in the post-MDT period needed CRRT. Eculizumab was well tolerated, and no adverse events were reported. Different authors have reported and suggested that early treatment with eculizumab is necessary to obtain successful outcomes when this treatment is used, even in those with secondary thrombotic microangiopathy [26–28]. The new workflow for the management of TMA allowed eculizumab treatment to be administered as soon as possible, and consequently, the optimal benefit was obtained.

An important finding in our study is that BMR performed by protocol showed different pathological findings in 4 patients in the post-MDT period, which confirmed that the central nervous system (CNS) is commonly affected in HUS patients. Traditionally, in TMA cases, TTP is suggested as the effect on the CNS, but our results indicated that BMR may be performed to identify extra-renal organ damage in HUS patients and to stratify the patient risk in a more precise manner. The CNS attachment to HUS has been described in shigatoxcin-related HUS, in whom the extent of BMR lesions did not correlate with clinical symptoms or worse prognosis [29, 30].

The retrospective design and the small number of patients included are the main weaknesses. Therefore, the present findings must be interpreted in the context of several limitations, while the main strength of our study is the difficult outcome evaluated.

## Conclusion

TMA is a severe disease or complication with multiple etiologies. The multidisciplinary team implemented in our hospital for its diagnosis and treatment has been associated with better outcomes, decreasing the risk of CRRT needs and lowering the RT and LOS. Moreover, the MDT better identified the organs affected in which especially useful is the utilization of BMR for accurately stratifying the risk of those patients. Consequently, the best treatment option could be recommended. The utilization of the online chat represents an important tool in the management of TMA to improve time and medical decisions in this potentially lethal disease. The implementation of the multidisciplinary team for the management and treatment of TMA should be considered a necessity not only to change the traditionally poor prognosis of the disease but also to reduce hospitalization length and improve understanding of this disease.

## Supporting information

S1 Table Raw data of the study impact of a multidisciplinary team for the management of thrombotic microangioapthy.xlsx”. (XLSX)
